# Co-existence of *KMT2A*::*SEPTIN6* fusion and *DIS3* variant in a pediatric case with acute myeloid leukemia: a case report and literature review

**DOI:** 10.3389/fonc.2023.1308786

**Published:** 2023-12-13

**Authors:** Liang Wang, Fangzhou Qiu, Yongming Shen, Sen Chen, Ping Si

**Affiliations:** ^1^ Department of Clinical Laboratory, Tianjin Children’s Hospital/Children’s Hospital, Tianjin University, Tianjin, China; ^2^ Department of Hematology, Tianjin Children’s Hospital/Children’s Hospital, Tianjin University, Tianjin, China

**Keywords:** acute myeloid leukemia, gene fusion, KMT2A, SEPTIN6, DIS3

## Abstract

The lysine(K)-specific methyltransferase 2A gene (*KMT2A*), previously known as mixed lineage leukemia (*MLL*), frequently rearranged in acute leukemia, belongs to one of the most promiscuous genes and has been found fused to more than 80 different partners. *KMT2A*::*SEPTIN6* fusion is a relatively uncommon rearrangement observed in pediatric acute myeloid leukemia (AML) patients, some of which may harbor other mutations. We herein report a case of AML-M4-infant with *KMT2A*::*SEPTIN6* fusion and *DIS3* variant. The 8-month-old girl presented with leukocytosis, anemia and thrombocytopenia. A bone marrow smear disclosed that 64% of the total nucleated cells were blasts. Karyotype analysis showed 46,X,t(X;11)(q24;q23)[10]/46,XX[10]. Fluorescence *in situ* hybridization analysis suggested a possible break in the *KMT2A* gene. After whole transcriptome sequencing, Exon 9 of *KMT2A* was fused in-frame with Exon 2 of *SEPTIN6*. This is a typical type of chromosomal rearrangement leading to the *KMT2A*::*SEPTIN6* fusion. Meanwhile, *DIS3* variant [c.2065C>T, p.R689X, variant allele frequency (VAF): 39.8%] was identified. *KMT2A*::*SEPTIN6* fusion has been associated with the pathogenesis of AML, whereas *DIS3* variants are relatively rare genetic events in pediatric AML. Regrettably, the relatives disagreed with the combination chemotherapy, and the patient eventually died of progressive disease. In conclusion, our findings provide a foundation for a better understanding of the genotypic profile of *KMT2A*::*SEPTIN6* associated AML, and the co-existence of *KMT2A*::*SEPTIN6* and *DIS3* variant might contribute to the disease progression and transformation of AML.

## Introduction

The rearrangements involving the lysine (K)-specific methyltransferase 2A gene (*KMT2A*), previously known as mixed lineage leukemia (*MLL*), have been found in more than 70% of the cases of infant leukemia, both acute myeloid leukemia (AML) or acute lymphoblastic leukemia (ALL) (1). The *KMT2A* gene located at 11q23, belongs to one of the most promiscuous genes and is fused to a variety of partner genes in acute leukemias. Up to now, a total of 107 in-frame *KMT2A* gene fusions have been identified, including *KMT2A*::*SEPTIN6* and *KMT2A*::*SEPTIN9* (2). *SEPTIN6*, located at Xq24, is highly conserved in eukaryotes and regulates various biological functions, including filament dynamics, cytokinesis and cell migration (3). Pediatric AML with *KMT2A*::*SEPTIN6* is very rare. To date, *KMT2A*::*SEPTIN6* has so far only been described in 18 pediatric AML in literature (4). The information on the clinical features and treatment strategies of such patients is limited.

DIS3 homolog, exosome endoribonuclease and 3’-5’ exoribonuclease (DIS3) is a highly conserved 3’ to 5’ exoribonuclease, which has a diverse range of functions within RNA metabolism including mRNA quality control, regulation of gene expression and small RNA processing (5). Whole genome sequencing in relapsed AML revealed that *DIS3*, as a recurrently mutated gene, was associated with AML relapse (6). Desterke et al. reported that *DIS3* missense mutations found in the cohort of AML patients affected VacB RNB protein domain, which was implicated in ribonuclease activity and RNA binding of the molecule (7). However, due to the rarity of *DIS3* variants in AML, the gene effect on leukemogenesis is still unknown. Here, we report the clinical and molecular characteristics of a *KMT2A*::*SEPTIN6* positive pediatric AML bearing *DIS3* variant, and reviewed the relevant literature and cases of AML to expand the current understanding of the genotypic spectrum of this rare form.

## Materials and methods

### Case presentation

The patient, an 8-month-old girl, visited the hospital due to a high-grade fever in June 2021, and a complete blood count showed that WBC (white blood cells): 26.34×10^9^/L (reference range: 5.00-14.20), PLT (platelets): 77×10^9^/L (reference range: 172-601), Hb (hemoglobin level): 99g/L (reference range: 103-138). Physical examination on admission revealed absence of hepatomegaly/splenomegaly or cutaneous lesions. A chest X-Ray was performed showing the inflammatory consolidation of lungs. Owing to the suspicion of acute leukemia, the patient was admitted to the hospital. After admission, laboratory tests confirmed the presence of anemia (Hb: 85g/L), leukocytosis (WBC: 28.2×10^9^/L) with 45% blasts on peripheral blood smear, and thrombocytopenia (PLT: 71×10^9^/L), as well as elevated serum inflammatory markers, including procalcitonin (0.09ng/ml, reference range: 0-0.05) and interleukin-6 (53.57pg/ml, reference range: 0-7.00). There was no evidence of coagulopathy or hyperuricemia. However, lactate dehydrogenase was elevated to 555 U/L (reference range: 120-300). The examinations of urine, stool and cerebrospinal fluid showed no significant abnormalities, and all blood culture tests were negative. A bone marrow (BM) smear disclosed a large number of immature cells and blast cells accounting for 64% of 200 nucleated cells. Cytochemistry analysis reported that most of the blast cells showed a positive reaction for peroxidase, and parts of them were weakly positive for sodium fluoride-sensitive alpha-naphthyl butyrate esterase. Immunophenotypic analysis revealed BM cells expressing HLA-DR, CD4, CD15, CD33, CD64 and MPO, mildly expressing CD11b, CD13, CD38 and CD117. A diagnosis of AML-M4 without neurological involvement was confirmed.

### Cytogenetic analysis

**Figure 1:**
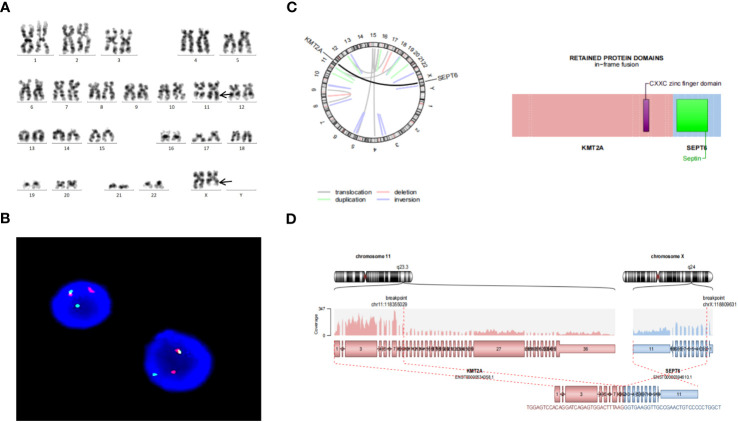
**(A)** Representative karyotype of the bone marrow cells was 46,X,t(X;11)(q24;q23)[10]/46,XX[10]. **(B)** FISH analysis using 11q23 break-apart probe. Separation of a green and a red signal revealed *KMT2A* rearrangement. **(C)** Circos plot indicating gene fusion between *KMT2A* and *SEPTIN6*. **(D)** *KMT2A* and *SEPTIN6* fusion transcripts. *KMT2A* Exon 9 was fused in-frame with *SEPTIN6* Exon 2.

The karyotype was found to be 46,X,t(X;11)(q24;q23)[10]/46,XX[10] (**Figure 1A**). Fluorescence *in situ* hybridization (FISH) was used for further understanding a variety of chromosomal abnormalities. FISH with break-apart 11q23 probe (Vysis, Abbott Molecular Inc.) confirmed *KMT2A* rearrangement showing the typical split signal (**Figure 1B**).

### Molecular analysis

According to the results of clinical diagnosis and cytogenetics, we carried out the whole transcriptome sequencing (WTS), which provided a comprehensive genomic profile. Peripheral blood in EDTA was collected for WTS detection. Ribo-zero based on probe hybridisation was used for depletion of rRNA. The RNA sequencing library was sequenced on the Illumina HiSeq X system, creating 19.55Gb total reads. The RNA-sequencing reads were mapped to the EnsemblGRCh37/hg19 by STAR aligner with the default parameters. The results revealed 5 fusion genes (*KMT2A*::*SEPTIN6, FUS*::*SETD1A, RBM4*::*SF1, ADRBK1*::*RBM4, EDF1*::*PBX3*), and 9 variants in following genes: *DIS3*, *ARID1A*, *SFPQ*, *THRAP3*, *EML4*, *AFF1*, *KMT2B*, *ASXL1* and *FLNA* (**Supplementary Tables S1**, **S2**).

The pathogenicity of the variants were assessed by the Association for Molecular Pathology (AMP), American Society of Clinical Oncology (ASCO), and College of American Pathologists (CAP) proposed standards and guidelines for the interpretation and reporting of sequence variants in cancer (8). Sequence variants were categorized into four categories based on their level of clinical significance: tier I, variants with strong clinical significance (level A and B evidence); tier II, variants with potential clinical significance (level C or D evidence); tier III, variants with unknown clinical significance; and tier IV, variants that are benign or likely benign. In this report, genetic analysis revealed that Exon 9 of *KMT2A* was fused in-frame with Exon 2 of *SEPTIN6* (**Figures 1C, D**). *KMT2A*::*SEPTIN6* fusion was categorized into tier I, which was pathogenic in AML. Furthermore, *DIS3* variant (c.2065C>T, p.R689X) was identified as a nonsense, leading to premature termination of protein coding. *DIS3* variant met the criteria for variants with Level C diagnostic/prognostic significance. Variants in this category include those that are diagnostic/prognostic for a group of related cancers or variants that are supportive of a diagnosis along with other genomic variants. *DIS3* variant was categorized into tier II, which was likely pathogenic in AML. However, other variants were categorized into tier III, which may have relevant pathological significance in further research.

### Treatment and outcome

Due to pneumonia, the patient received intravenous latamoxef sodium (40 mg/kg/day, every 12 h) for anti-infection therapies. The patient was treated with cytoreductive therapy of hydroxyurea to prevent leukocytosis. After 5 days of hospitalization, WBC level dropped to the normal reference value with no metabolic manifestation observed (no evidence for tumor lysis syndrome) and 12% blasts detected in peripheral blood. Because the relatives disagreed with the combination chemotherapy, the patient did not receive medication, and the patient eventually died of progressive disease.

### Literature review

PubMed database was searched with keywords including acute myeloid leukemia, *KMT2A*::*SEPT6*, *MLL*::*SEPT6*, *KMT2A*::*SEPTIN6*, *MLL*::*SEPTIN6* and *DIS3* to gather related case reports. To date, *KMT2A*::*SEPTIN6* has only been reported in 28 pediatric cases with AML, including one case in present study and twenty-seven cases from the literature (4, 9–24) (**Table 1**). Clinical information and genetic features of evaluable *KMT2A*::*SEPTIN6*-positive pediatric cases with AML are summarized in **Table 1**. The age of the patients ranged from 0 to 10 years, with a male-female ratio of 1.8:1 (18 males vs. 10 females). All patients were diagnosed with AML according to the former FAB classification: one child with M1, nine children with M2, one child with M3, eight children with M4, six children with M5, and 3 children unknown. Most of them had available cytogenetic information, and complex chromosomal karyotypes with diverse chromosomal abnormalities, including chromosomal translocations (8 patients), chromosomal insertions (8 patients) and chromosomal complex abnormalities (11 patients). As for the treatment, most of them received chemotherapy or bone marrow transplantation (BMT). Ten patients died. AML with *DIS3* variants is rare. Among the 108 patients of M1 and M3 AML, 3 cases had the *DIS3* variants (25). In 193 patients with AML, 2 patients with AML-M1 presenting a co-association of *RUNX1* and *DIS3* mutations were found, and one patient with AML-M3 presented an isolated *DIS3* variant without co-association with a *RUNX1* mutation (8). Furthermore, it has been reported that 8 different types of *DIS3* variants were described (26) (**Table 2**).

**Table 1:** Previous reports and the present case of pediatric AML with *KMT2A*::*SEPTIN6* fusion.

Case	Sex	Age	WBC (×10^9^/L)	PLT (×10^9^/L)	Hb (g/L)	FAB	Karyotype	Fused Exon	Treatment protocol	Survival outcome	Reference
1	M	3y	NA	NA	NA	M2	46,Y,t(X;11)(q22;q23)	NA	BMT	Alive	(9)
2	M	1y	NA	NA	NA	M4	46,Y,t(X;11)(q13;q23)	NA	BMT	Died	(9)
3	M	1y	NA	NA	NA	M5	46,Y,t(X;11)(q24;q23)	NA	Chemotherapy	Alive	(9)
4	M	6y	1.60	254	86	NA	46,XY,t(X;11)(q24;q23)	NA	At diagnosis: doxorubicin, cytarabine, methotrexate, vincristine, and 6-mercaptopurine. At relapse: all-trans retinoic acid, G-CSF, cytarabine,etoposide, and Mitoxantron.	Died	(10)
5	F	6m	280.00	NA	NA	M2	46,XX,ins(X;11)(q24;q23)	Exon 8/Exon 2	AML-BFM 98 protocol	Alive	(11)
6	M	10m	13.40	NA	NA	M2	46,Y,t(X;11)(q22;q23)[25]/46,XY[5]	Exon 8/Exon 2	CCG 2891 protocol + haploidentical transplant	Alive	(12)
7	F	20m	397.00	NA	NA	M4	47,X,der(X) t(X; 11) (q22; q23) t (3;11)(p21; q12), der (3) t(3;11) (p21;q23)t(X;11)(q22;q25), + 6, der(11)del(11)(q12?qter)	Exon 7/Exon 2	Chemotherapy	Died	(12)
8	F	3m	163.20	NA	NA	M2	46,XX,t(5;11)(q13;q23)[6]/46,sl,add(X)(q22)[12]/47,sdl, +add(X)[1]/46,XX[1]	Exon 7/Exon 2; Exon 8/Exon 2	Chemotherapy +BMT	Died	(13)
9	M	7m	608.00	NA	NA	M2	46,XY[20]46,XY,ins(X;11)(q22-q24 ;q23)	Exon 7/Exon 2	Chemotherapy +BMT	Alive	(13)
10	F	6m	58.50	NA	NA	M1	46,X,add(X)(q2?),del(11q?)[20]	Exon 7/Exon 2; Exon 8/Exon 2	Chemotherapy +BMT	Died	(13)
11	M	8m	112.00	41	84	M4	46,Y,ins(11; X)(q23;q24q24)	Exon 8/Exon 2	1-β-Darabinofurano sylcytosine + daunomycin + etoposide +intrathecal chemotherapy	Died	(14)
12	M	29m	NA	NA	NA	M5	46,Y,ins(X;11)(q24;q23q13)[13]/46,XY[7]	Exon 7/Exon 2	BHAC regimen	Alive	(15)
13	M	13m	35.00	35	64	M4	46,Y,ins(11;X)(q23;q24q22) [14]/46,idem,i (10)(q10)	Exon 9 / Exon 2	CCG 2891 protocol, Regimen C +intensive Intrathecal chemotherapy + radiation to the Extramedullary sites of disease +BMT	Alive	(16)
14	F	26m	NA	NA	NA	M4	46,XX,t(11;17)(q23;q?25)[20]	Exon 7/Exon 2; Exon 8/Exon 2	NA	NA	(17)
15	M	0.7y	NA	NA	NA	M4	46,XY	Exon 8/Exon 2	NA	NA	(18)
16	F	17m	NA	NA	NA	M2	47,X,add(X)(p11),+6,add (11) (q23)[20]	Exon 7/Exon 2; Exon 8/Exon 2; Exon 5-7/Exon 2	NA	NA	(19)
17	M	0m	NA	NA	NA	NA	46,Y,ins(X;11)(q24;q13q23)[11]	Exon 7/Exon 2; Exon 5-7/Exon 2	NA	NA	(19)
18	M	12m	NA	NA	NA	NA	46,Y,t(X;11)(q24;q23)[11])/46,XY[9]	Exon 6/Exon 2; Exon 5/Exon 2	NA	NA	(19)
19	F	1y	16.40	81	93	M2	47,X,add(X)(p11),+6,add (11) (q23)[20].ish der(X)add(X)(p11)ins(X;11) (q?;q23q23)(5′MLL+),der (11)ins(11;?)(q22;?)ins(X;11)(5′MLL−,3′MLL+)	Exon 10/Exon 2; Exon 11/Exon 2	ELAM 02 + BMT	Alive	(20)
20	M	1y	NA	NA	NA	M5	46,Y,der(X)t(X;11),der (11)t (X;11)(?;?p14)t(X;11)(?;q23)t (X;12)(?;q?13),del (12)(q?22),der (12)t (X;12)/47,idem,+19	NA	NA	NA	(21)
21	F	5y	NA	NA	NA	M5	49,X,ins(X;11)(q24;q13q23),+4,+8,+20	NA	NA	NA	(21)
22	M	10y	3.70	245	114	M2	46,Y,-X,del (6)(q23),add (11) (q23),add (22)(q13),+mar[13]/46, XY[7]	NA	Chemotherapy +BMT	Alive	(22)*(Preprint)*
23	M	12m	73.35	67	89	M5	46,Y,ins(X;11)(q24;q23q13) [12]/46,XY[3]	NA	ELAM 02	Alive	(4)
24	M	0m	112.00	9	90	M5	46,Y,ins(X;11)(q23;q24q12)[10]	NA	No chemotherapy	Died	(23)
25	M	16m	123.80	39	41	M4	46,Y,t(X;11)(q24;q23),del[7] (q21q31)[13]/46,XY[2]	NA	HA regimen	Died	(23)
26	M	9y	3.00	245	93	M2	45,Y,del(X)(q21),der[11]t(X;11)(q24;q23),-20,add[22](q13)[3]	NA	MAE regimen + intrathecal chemotherapy +BMT	Alive	(23)
27	F	3y	7.10	NA	NA	M3	46,XX, ins(X;11)(q24;q14q25)	Exon 10/Exon 2; Exon 11/Exon 2	all-trans retinoic acid, cytarabine,daunorubicin	Died	(24)
28	F	8m	28.20	71	85	M4	46,X, t(X;11)(q24;q23)[10]/46,XX[10]	Exon 9 / Exon 2	No chemotherapy	Died	Present study

M, male; F, female; m, months; y, years; BMT, bone marrow transplantation; AML-BFM, acute myeloid leukemia-Berlin-Frankfurt-Munster; CCG, the Children’s Cancer Group; BHAC, idarubicin + 1-β-D-arabinofuranosylcytosine + thioguanine; HA, homoharringtonine + cytarabine; MAE, Mitoxantrone + cytarabine + etoposide; NA, not available.

**Table 2:** Summary of characteristics of *DIS3* variants in AML.

ID	NM	Exon	Mutation	Reference allele	Alternative allele	Type of mutation
rs149628103	NM_001128226:c.533T>C	Exon4	I178T	A	G	Missense
rs4883918	NM_001128226:c.716A>G	Exon5	N239S	T	C	Missense
rs7332388	NM_001128226:c.887C>G	Exon6	T296R	G	C	Missense
rs149755140	NM_001128226:c.1042A>G	Exon8	K348E	T	C	Missense
rs35288597	NM_001128226:c.1222G>A	Exon9	D408N	C	T	Missense
rs2196979	NM_001128226:c.1677G>A	Exon14	T559	C	T	Silent
rs35017269	NM_001128226:c.1813C>T	Exon15	P605S	G	A	Missense
rs35036619	NM_001128226:c.2443A>G	Exon19	I815V	T	C	Missense

## Discussion

AML with *KMT2A* abnormalities represent a subset of pediatric leukemia. Five different *SEPTIN* genes have been identified as *KMT2A* fusion partners, giving rise to chimeric fusion proteins. Among them, *KMT2A*::*SEPTIN6* is often complex and sometimes cryptic as a result of the opposite orientation of *KMT2A* and *SEPTIN6* on the respective chromosome arms. *KMT2A*::*SEPTIN6* fusion gene was, to our knowledge, described in some children and very few adult cases diagnosed with AML (5, 23). In this study, we described a pediatric AML with *KMT2A*::*SEPTIN6* fusion. In order to improve the understanding of this rare form of pediatric AML, we reviewed the relevant literature and summarized the characteristics of *KMT2A*::*SEPTIN6*-positive cases.

**Table 1** lists the detailed clinical features, treatment strategies and prognosis of evaluable patients. Among these cases, nearly 60% were infant patients (≤ 1 year old). It is worth noting that 5 (50%) of the 10 deceased patients were infants. These findings suggest that age is an important factor affecting survival time. Most patients had typical characteristics of leukemia, such as leukocytosis, anemia and low platelet counts. Chen et al. reported that the overall survival of patients with WBC levels ≥ 20.0 ×10^9^/L was much shorter than that of patients with WBC levels < 20.0 ×10^9^/L in *KMT2A*::*SEPTIN6* cases (23). An interesting finding was that, out of the 25 evaluable cases diagnosed with AML according to the former FAB classification, 14 (56%) were classified as FAB M4 or M5 subtypes (AML-M4/M5). Consistent with previous studies, AML-M4/M5 was frequently associated with *KMT2A* gene rearrangement and its incidence was relatively high among infants (27). In addition to the translocations and insertions, the complex karyotypes were observed in pediatric AML with *KMT2A*::*SEPTIN6* fusion, and there were additional chromosomal abnormalities, such as add, del, der, idem, +4, +6, +8, +10, +19 and +20. Sequencing analysis demonstrated that fusions between *SEPTIN6* Exon 2 and *KMT2A* Exon 7, 8 or both of them to be the most frequent. The fusion between *KMT2A* Exon 9 and *SEPTIN6* Exon 2, as in our study, was a rare occurrence. These acquired genetic abnormalities can play an essential role in the pathogenesis.

There is a lack of systematic evaluation on the prognosis of *KMT2A*::*SEPTIN6*-associated AML. It was reported that a pediatric AML with the *KMT2A*::*SEPTIN6* fusion initially responded well to multiagent chemotherapy, but a hematologic relapse occurred 2 months later (14). Similarly, the previously published AML-M4 case with *KMT2A*::*SEPTIN6* fusion, received chemotherapy and had complete remission at 8 weeks of treatment; however, one month following the end of therapy, the patient developed a testicular relapse and presented recurrence of the cytogenetic abnormality (16). These results implicate that *KMT2A*::*SEPTIN6*-associated AML is easy to relapse and has a poor prognosis. Due to the rarity of this form, the pathogenesis of *KMT2A*::*SEPTIN6* associated AML is not clear. Santos et al. found that a statistically significant down-regulation was observed for the RNA expression of both *KMT2A* and *SEPTIN6* in *KMT2A*::*SEPTIN6* leukemia (28). SEPTIN6 was the component of a core septin hexamer complex (SEPTIN2-SEPTIN6-SEPTIN7 complex), whose formation was thought to be essential to proper cytokinesis. And the correct expression of SEPTIN2, SEPTIN6, and SEPTIN7 seemed to be also relevant for the correct functioning of the cell DNA damage checkpoint (29). These results suggest that a link between aberrant septin expression and deregulation of the cell-cycle machinery is a crucial physiologic cellular mechanism of leukemogenesis. In addition, the fusion partners appeared to convert the rearranged KMT2A protein to a potent transcriptional activator. *KMT2A* rearrangements resulted in deregulation of KMT2A protein activity causing abnormal patterns of class I homeobox gene expression in hematopoietic stem cells or progenitors, thus promoting leukemogenesis. However, *in vitro* and *in vivo* models of the *KMT2A*::*SEPTIN6* fusion have indicated that *KMT2A*::*SEPTIN6* by itself was able to induce lethal myeloproliferative disease but not to induce acute leukemia in mice, implying that secondary genotoxic events related to DNA repair or cell cycle regulation could be required to develop leukemia (30).

The *DIS3* gene located at chromosome 13q22.1, encods for a highly conserved ribonuclease indispensable for survival in vertebrates. *DIS3* variants have been associated with multiple myeloma. Todoerti et al. reported that the frequency of *DIS3* variants in newly diagnosed multiple myeloma was approximately 10% (31). But *DIS3* variants have rarely been described in AML. 8 variants in *DIS3* gene were previously described in AML (**Table 2**). The types of *DIS3* variants included nonsense, missense and silent. These disruptive events lead to the loss of full-length DIS3 protein and to the lack or low expression of a truncated form of the protein, suggesting an oncogenic potential for *DIS3* variants. Furthermore, *DIS3* variants were associated with an important down-regulation of genes involved in the cohesin complex and a down-regulation of molecules implicated in DNA double strand repair in M1 patients with double *DIS3-RUNX1* mutations, which were thought to indicate an important loss of control for the entry in mitosis (7). These findings indicates that *DIS3* variants may play an important role in transformation and progression of AML.

## Conclusion

In conclusion, we have described a rare pediatric case with *KMT2A*::*SEPTIN6* associated AML, in particular, bearing *DIS3* variant. Since reports of pediatric case are limited, it is difficult to deduce any conclusions involving the prognostic significance of this form. The potential effects require further screening of a large cohort of AML patients with both aberrations. This case extends the mutational spectrum, providing an opportunity to broaden the understanding available of this rare form of AML. Further functional studies are required to better elucidate the pathogenesis of this disease.

## Data availability statement

The datasets presented in this study can be found in online repositories. The names of the repository/repositories and accession number(s) can be found in the article/**Supplementary Material**.

## Ethics statement

The studies involving humans were approved by the Medical Ethics Committee of Tianjin Children’s Hospital. The studies were conducted in accordance with the local legislation and institutional requirements. Written informed consent for participation in this study was provided by the participants’ legal guardians/next of kin. Written informed consent was obtained from the individual(s), and minor(s)’ legal guardian/next of kin, for the publication of any potentially identifiable images or data included in this article.

## Author contributions

LW: Writing – original draft. FQ: Writing – original draft. YS: Writing – original draft. SC: Writing – review & editing. PS: Writing – review & editing.
