# Supplementary material for: Co-existence of KMT2A::SEPTIN6 fusion and DIS3 variant in a pediatric case with acute myeloid leukemia: a case report and literature review
Source: Front Oncol. 2023 Dec 13;13:1308786. doi: 10.3389/fonc.2023.1308786 (PMC10751303; doi:10.3389/fonc.2023.1308786)
Supplement: Supplementary file 1 [file DataSheet_1.doc]

**Supplemental TABLE 1 The fusion genes of pediatric AML in this study.**

| **Gene 1** | **Gene 2** | **Position 1** | **Position 2** | **Transcript strand 1** | **Transcript strand 2** | **Type of**  **fusion** |
| --- | --- | --- | --- | --- | --- | --- |
| *KMT2A* | *SEPTIN6* | chr11:118355029 | chrX:118809631 | + | - | translocation |
| *FUS* | *SETD1A* | chr16:31191548 | chr16:30970038 | + | + | duplication |
| *RBM4* | *SF1* | chr11:66384528 | chr11:64544098 | + | - | inversion |
| *ADRBK1* | *RBM4* | chr11:67034283 | chr11:66407171 | + | + | duplication |
| *EDF1* | *PBX3* | chr9:139757740 | chr9:128510829 | - | + | inversion |

**Supplemental TABLE 2 The variants of pediatric AML** **in this study.**

| **Gene** | **Transcript** | **Exon** | **Allele change** | **Mutation** | **VAF (%)** |
| --- | --- | --- | --- | --- | --- |
| *DIS3* | NM_014953 | Exon16 | c.2065C>T | R689X | 39.8% |
| *ARID1A* | NM_006015 | Exon1 | c.461A>C | Y154S | 7.7% |
| *SFPQ* | NM_005066 | Exon3 | c.1169C>G | A390G | 57.6% |
| *THRAP3* | NM_005119 | Exon3 | c.43C>T | R15C | 38.2% |
| *EML4* | NM_019063 | Exon13 | c.1441A>G | I481V | 37.8% |
| *AFF1* | NM_001166693 | Exon14 | c.2746G>T | D916Y | 54.0% |
| *KMT2B* | NM_014727 | Exon3 | c.1095A>C | E365D | 4.2% |
| *ASXL1* | NM_015338 | Exon12 | c.2779G>A | G927R | 48.3% |
| *FLNA* | NM_001110556 | Exon22 | c.3325G>A | E1109K | 94.0% |
